# Smoking in the home after childbirth: prevalence and determinants in an English cohort

**DOI:** 10.1136/bmjopen-2015-008856

**Published:** 2015-09-08

**Authors:** Sophie Orton, Tim Coleman, Laura L Jones, Sue Cooper, Sarah Lewis

**Affiliations:** 1 UK Centre for Tobacco & Alcohol Studies & Division of Primary Care, University of Nottingham, Nottingham, UK; 2 UK Centre for Tobacco & Alcohol Studies & Unit of Public Health, Epidemiology & Biostatistics, School of Health & Population Sciences, University of Birmingham, Birmingham, UK; 3 UK Centre for Tobacco & Alcohol Studies & Division of Epidemiology & Public Health, University of Nottingham, Nottingham, UK

**Keywords:** PAEDIATRICS, PRIMARY CARE, PUBLIC HEALTH

## Abstract

**Objectives:**

Children's exposure to secondhand smoke (SHS) is causally linked to childhood morbidity and mortality. Over 38% of English children (aged 4–15) whose parents are smokers are exposed to SHS in the home. Little is known about the prevalence of SHS exposure in the homes of young infants (≤3 months). This study aimed to estimate maternal self-reported prevalence of SHS exposure among infants of women who smoked just before or during pregnancy, and identify factors associated with exposure.

**Setting:**

Primary Care, Nottingham, England.

**Participants:**

Current and recent ex-smoking pregnant women (n=850) were recruited in Nottingham, England. Women completed questionnaires at 8–26 weeks gestation and 3 months after childbirth. Data on smoking in the home 3 months after childbirth was available for 471 households.

**Primary and secondary outcome measures:**

Maternal-reported smoking in the home 3 months after childbirth.

**Results:**

The prevalence of smoking in the home 3 months after childbirth was 16.3% (95% CI 13.2% to 19.8%) and after multiple imputation controlling for non-response 18.2% (95% CI 14.0% to 22.5%). 59% of mothers were current smokers; of these, 24% reported that smoking occurred in their home compared to 4.7% of non-smokers. In multivariable logistic regression, mothers smoking ≥11 cigarettes per day were 8.2 times (95% CI 3.4 to 19.6) more likely to report smoking in the home. Younger age, being of non-white ethnicity, increased deprivation and less negative attitudes towards SHS were also associated with smoking in the home.

**Conclusions:**

This survey of smoking in the home 3 months after childbirth found a lower prevalence than has been reported in older children. Interventions to support smoking mothers to quit, or to help them restrict smoking in the home, should target attitudinal change and address inequality relating to social disadvantage, younger age and non-white ethnic groups.

**Strengths and limitations of this study**

- This is the first survey since the introduction of smoke-free legislation, as far as we are aware, of smoking in the home immediately after childbirth.
- During recruitment, 96% of women attending selected antenatal clinics within Nottingham University Hospital Trust were screened for eligibility, accounting for around one-third of all births within Nottingham, England, during this time.
- The demographic profile of smokers within this cohort is similar to other UK pregnancy cohorts, meaning the sample is likely to be broadly representative.
- A potential limitation was the reliance on reported smoking measures.
- There were some differences between those who responded and those who did not respond at follow-up, however appropriate imputation methods were used to allow for this non-response bias.

## Background

Exposure to secondhand smoke (SHS) is the involuntary inhalation of other people's cigarette smoke and globally, 40% of children are exposed.^1^ Children's SHS exposure has been causally linked to respiratory tract infections, middle ear disease, sudden unexplained death in infancy and asthma.^2^ The WHO believes that SHS is a substantial threat to child health,^3^ and the US Surgeon General argues there is no safe exposure level.^4^

In 2008, a study conducted in England reported 52% of children aged 4–15 whose parents were smokers were exposed to SHS in the home.^5^ This has reduced in recent years, with a reported 38.7% of children of smoking parents aged 4–15 years being exposed to SHS in the home in England in 2012,^6^ however it clearly remains a significant problem. Similar trends have been observed elsewhere, both in the UK,^7–10^ and internationally (eg, USA;^11^ ^12^ Ireland, France, Germany and the Netherlands 13). However, current UK prevalence estimates for children's SHS exposure in the home focus on children aged >4 years,^6–10^ ^14–16^ and most studies include children aged 10–11 years.^7–10^ ^16^ There is therefore little research examining SHS exposure in the home among young infants (≤3 months) and few prevalence estimates. We are aware of only one UK study estimating the prevalence of SHS among young infants. Among children of smokers, 82% of infants (average age 3 months) experienced SHS exposure in the home.^17^ Elsewhere, we are aware of just two studies, from the USA, in which 10.8–21.4% of infants of smoking mothers aged ≤9 months were exposed to SHS in the home^18^ and 24.5% were exposed to SHS for ≥1 h per day.^19^ Although these studies suggest SHS exposure may be a substantial issue, they were conducted prior to,^17^ ^19^ or around the time^18^ that comprehensive smoke-free legislations were introduced. There are no contemporary estimates of prevalence in this age group.

Additionally, of 41 studies investigating factors associated with children's SHS exposure in the home identified by systematic review,^20^ only three^19^ ^21^ ^22^ included infants or children aged <2 years. This review found parental smoking, low socioeconomic status (SES) and being less educated were all consistently independently associated with children's SHS exposure in the home.^20^ However, due to the small number of studies focusing on younger age groups, little is known about the influences on SHS exposure in the home experienced by young infants; consequently, this paper reports the prevalence of SHS exposure among young infants born to women from an English pregnancy cohort, and identifies factors associated with this exposure.

## Methods

This study presents secondary analysis on data collected as part of the longitudinal cohort, the Pregnancy Lifestyle Survey (PLS); methods and cohort characteristics have been described in detail previously.^23^ The study received a favourable opinion from Derbyshire Research Ethics Proportionate Review Sub-Committee (reference 11/EM/0078).

### Participants

The baseline sample size for the PLS was 850, based on the cohort's primary aim to estimate the proportion of smokers who initiate quit attempts in the second or third trimester of pregnancy.^23^ Women who were aged ≥16 years, between 8 and 26 weeks pregnant, and self-reported being current smokers or having smoked in the 3 months prior to pregnancy were eligible for participation.

### Recruitment and data collection

Participants were recruited between August 2011 and August 2012 at two antenatal clinics within Nottingham University Hospitals NHS Trust, England. Participants completed a baseline questionnaire in the antenatal clinic when they were between 8 and 26 weeks gestation, and a follow-up questionnaire when their baby was 3 months old. At Follow-up, hospital administration staff obtained participants’ delivery dates from their antenatal records. Participants were sent a questionnaire 3 months after their delivery date by post or email; if not returned, completion by telephone was attempted.

The questionnaires have been described elsewhere.^23^ In summary, both the baseline and the follow-up questionnaires were similar in format and content, using yes/no, multiple choice and five-point Likert items. The baseline questionnaire was divided into six sections: screening questions, health and pregnancy, smoking beliefs, current smoking behaviour, interest in getting help to stop smoking and sociodemographic information. At the 3-month follow-up, the same topics were covered but edited to reflect women's postnatal status. Additional questions about smoking in the home, and beliefs about harm caused to infants and children through SHS exposure were included.

### Outcomes

The primary outcome measure was maternal-reported smoking by either themselves or someone else in their home 3 months after childbirth, using participants’ responses to the questions ‘how often do you smoke in your home nowadays?’ and ‘how often do other people smoke in your home nowadays?’. Responses used Likert items ranging from 1 (‘never’) to 5 (‘very often’). A binary outcome was created, where participants who responded ‘almost never’ to ‘very often’ (2–5 on scale) to either of these questions were considered to have smoking in the home 3 months after childbirth, and participants who responded ‘never’ to have a smoke-free home.

The maternal sociodemographic characteristics of age, ethnicity, highest qualification, age left full-time education and current employment status were taken from baseline questionnaires. Age left full-time education was categorised as ≤16 years (UK age of compulsory education), ≥16 years and still in full-time education. Ethnicity was categorised as a binary variable (white British vs other ethnicity) due to small numbers of participants in non-white British ethnic groups. A measure of socioeconomic status (SES) was created by mapping participants’ postcodes with corresponding 2007 Indices of Multiple Deprivation (IMD) scores, taken from routine UK Data Service data.^24^ The 2007 IMD measures a range of domains reflecting economic, social and housing issues, where higher scores reflect greater deprivation. Scores were divided into tertile groups.

Participant's self-reported smoking behaviour was measured at both time points. Women were categorised as being a non-smoker, or smoking 0–5, 6–10, ≥11 cigarettes per day. Heaviness of smoking index (HSI) scores were calculated using the method described by Borland *et al*^25^ Partner smoking status at 3 months after childbirth was categorised as non-smoker, smoker or not applicable/no partner.

**Figure 1:**
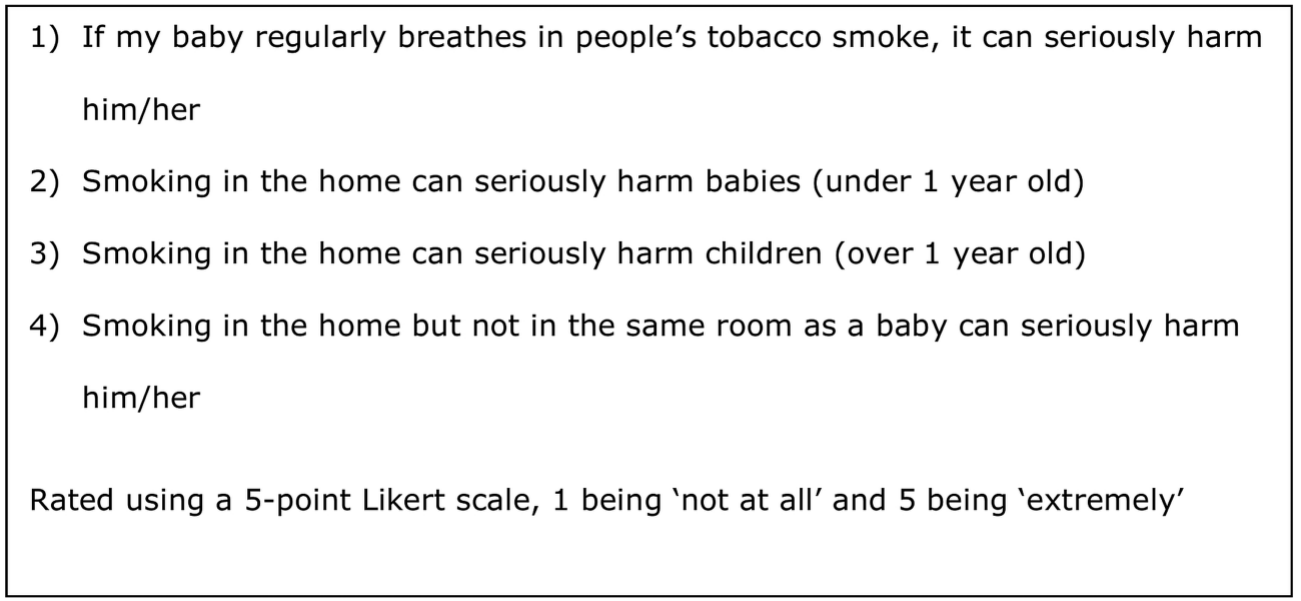
Attitudes to child secondhand smoke exposure scale items.

Attitudes to children's SHS exposure were measured by asking participants the extent to which they agreed with four attitudinal statements using Likert items (figure 1). The items had high-internal consistency (Cronbach's α=0.9),^26^ and so responses were combined into a single summed score (out of 20), whereby a higher score reflected a more negative attitude towards children's SHS exposure. Attitude scores were highly negatively skewed, and so were categorised into a binary variable; a score of ≥15 represented ‘negative attitudes towards child SHS exposure’ and a score of <15 ‘less negative attitudes towards child SHS exposure’.

### Data analysis

Statistical analyses were conducted using Stata V.13.^27^ Cohort characteristics and the characteristics of responders and non-responders at 3 months after childbirth are presented, and differences examined using χ^2^ tests for categorical data and t tests for continuous data. The prevalence of smoking in the home was estimated using those with complete data; given that there was a high level (50%) of missing data at 3 months after childbirth and observed differences in the characteristics of responders and non-responders, multiple imputation methods^28^ were used to impute values for missing outcome data. Five imputed data sets were considered sufficient^29^ and were constructed using the mi command in Stata, based on the following baseline variables: smoking behaviour, HSI, age, ethnicity, qualifications, employment, IMD score and partner smoking status. These variables were selected based on characteristics associated with child SHS exposure in the home in a previous systematic review^20^ and variables associated with non-response.^30^ The imputed outcome variable was only used for estimates of prevalence of smoking in the home; all other analyses were conducted using the original non-imputed outcome variable.

The variables smoking behaviour at baseline, smoking behaviour 3 months after childbirth, age, ethnicity, highest qualification, age left full-time education, employment status, IMD, partner smoking status and attitude towards child SHS exposure score were entered into a univariate logistic regression analysis and the ORs and 95% CI calculated. For continuous exposure variables, the linearity of the effect was tested using the likelihood-ratio test.

Those variables that were statistically significant in univariate analysis at the p<0.05 level, or with strong a priori assumptions (eg, maternal education) based on the findings of a systematic review,^20^ were entered into exploratory multivariable logistic regression models. Correlations were observed between smoking behaviour at baseline, smoking behaviour at 3 months after childbirth and baseline HSI. Smoking behaviour at 3 months after childbirth was most strongly associated with the outcome measure, and was therefore included in the multivariable analyses and the other smoking variables omitted to avoid collinearity. Similarly, highest qualification and age left full-time education were considered in the multivariable analysis independently due to collinearity. Those variables reaching significance (p<0.05) were retained in the model, and non-significant variables re-entered into the model sequentially. Participants with missing data for exposure variables were excluded from multivariable analysis (n=6). ORs, 95% CI, and likelihood ratio test p values and Wald's p values for trend for ordered categorical exposure variables are reported.

## Results

### Cohort characteristics

The cohort consisted of 850 pregnant women, of which 56.6% were current smokers at baseline (table 1). The demographic profile of smokers within the cohort was similar to other UK pregnancy cohorts.^23^

**Table 1:** Cohort characteristics and comparison between responders and non-responders at 3 months postnatal

Characteristic	All cohort N (%)	Responders at 3 months postnatal N (%)	Non-responders and withdrawals at 3 months postnatal N (%)	p Value
	N=850	N=476	N=374	
Smoking behaviour baseline				
Recent ex-smoker	362 (43.4)	235 (50.1)	127 (34.7)	
≤5 cigarettes per day	191 (22.9)	105 (22.4)	86 (23.5)	
6–10 cigarettes per day	151 (18.08)	71 (15.1)	80 (21.9)	
≥11 cigarettes per day	131 (15.4)	58 (12.4)	73 (20.0)	<0.0001
Age (years) Mean (SD)	25.8 (5.5)	26.5 (5.6)	24.8 (5.3)	<0.0001
Ethnicity				
White British	751 (89.0)	421 (89.0)	330 (89.0)	
Other ethnicity	93 (11.0)	52 (11.0)	41 (11.1)	0.0007
Highest qualification				
No qualifications	155 (18.2)	62 (13.0)	94 (25.1)	
GCSEs or equivalent	355 (41.8)	184 (38.7)	171 (45.7)	
AS/A-Levels or equivalent	174 (20.5)	118 (24.8)	56 (15.0)	
Degree or equivalent	133 (15.7)	95 (20.0)	38 (10.2)	
Other qualification	33 (2.9)	17 (3.6)	16 (4.3)	<0.0001
Age left education				
≤16 years of age	469 (56.4)	232 (50.0)	237 (64.6)	
≥17 years of age	334 (40.2)	211 (45.4)	123 (33.5)	
Still in full-time education	28 (2.4)	21 (4.5)	7 (1.9)	<0.0001
Employment				
Paid work, manual	158 (18.7)	102 (21.5)	56 (15.0)	
Paid work, non-manual	180 (21.3)	131 (27.6)	49 (13.1)	
Paid work, unclear whether manual/non-manual	45 (5.3)	27 (5.7)	18 (4.8)	
Unemployed	201 (23.7)	92 (19.4)	109 (29.2)	
Full-time parent	219 (25.9)	97 (20.5)	122 (32.7)	
Full-time student	23 (2.7)	13 (2.7)	10 (2.7)	
Other	21 (2.5)	12 (2.5)	9 (2.4)	<0.0001
Indices Multiple Deprivation score (IMD)*				
1st tertile	284 (33.6)	178 (37.4)	106 (28.8)	
2nd tertile	279 (33.1)	162 (34.0)	117 (31.8)	
3rd tertile	281 (33.3)	136 (28.6)	145 (39.4)	0.002
Baseline heaviness of smoking index (smokers only)				
Low addiction	321 (67.6)	171 (72.8)	150 (62.5)	
Moderate addiction	146 (30.7)	61 (26.0)	85 (35.4)	
High addiction	8 (1.7)	3 (1.3)	5 (2.1)	0.06
Partner smoking baseline				
Partner does not smoke tobacco	499 (59.1)	172 (36.4)	122 (32.9)	
Partner smokes tobacco	294 (34.8)	279 (58.9)	220 (59.3)	
No partner	51 (6.0)	22 (4.7)	29 (7.8)	0.12

*Higher score reflects greater deprivation.

GCSE, General Certificate of Secondary Education.

### Follow-up response rates

**Figure 2:**
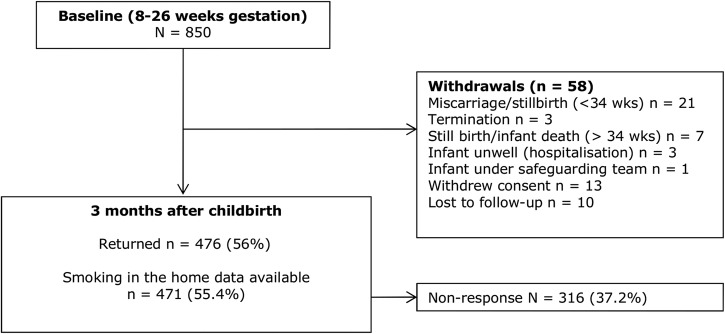
Consort diagram of response rates and reasons for withdrawal.

At follow-up, the response rate was 56% (n=476) after non-response and withdrawal (figure 2). Owing to missing data in some of the returned questionnaires, smoking in the home information was available for 471 participants. Table 1 shows the characteristics of women who did and did not respond to the follow-up questionnaire 3 months after childbirth.

### Smoking in the home 3 months after childbirth: prevalence and determinants

The ‘raw’ prevalence of smoking in the home 3 months after childbirth was 16.3% (95% CI 13.2% to 19.8%). At this time, 59% of mothers were current smokers; of these, 24% reported smoking in the home compared to 4.7% of non-smokers (table 2). After controlling for non-response bias using multiple imputation methods, prevalence of smoking in the home 3 months after childbirth was 18.2% (95% CI 14.0% to 22.5%).

**Table 2:** Prevalence of smoking in the home, and univariate and multivariable analysis of associated factors

Characteristic	All sample	Smoking occurs in the home	Unadjusted OR	Adjusted OR
	(N=471)	(N=76)		(N=465)
	N (column %)	N (row %)	OR (95% CI)	OR (95% CI)
Smoking status baseline				
Recent ex-smoker	231 (49.0)	20 (8.7)	Reference*	
Current smoker	240 (51.0)	56 (23.3)	3.21 (1.86 to 5.60)	
Smoking behaviour baseline				
Recent ex-smoker	231 (49.8)	20 (8.7)	Reference*	
≤5 cigarettes per day	104 (22.4)	19 (18.3)	2.36 (1.20 to 4.64)	
6–10 cigarettes per day	71 (15.3)	14 (19.7)	2.60 (1.23 to 5.45)	
≥11 cigarettes per day	58 (12.5)	21 (36.2)	5.99 (2.96 to 12.12)	
Smoking status 3 months after childbirth				
Ex-smoker	192 (40.8)	9 (4.7)	Reference*	
Current smoker	279 (59.2)	67 (24.0)	6.43 (3.12 to 13.25)	
Smoking behaviour 3 months after childbirth				
Ex-smoker	192 (40.8)	9 (4.7)	Reference*	Reference*
≤5 cigarettes per day	105 (22.3)	25 (23.8)	6.35 (2.84 to 14.23)	6.17 (2.63 to 14.46)
6–10 cigarettes per day	83 (17.6)	11 (13.3)	3.11 (1.24 to 7.81)	2.09 (0.78 to 5.63)
≥11 cigarettes per day	91 (19.3)	31 (34.1)	10.51 (4.73 to 23.32)	8.17 (3.41 to 19.55)
Baseline heaviness of smoking index				
Low addiction	171 (36.3)	35 (20.5)	Reference*	
Moderate addiction	60 (12.7)	17 (28.3)	1.54 (0.78 **to** 3.01)	
High addiction	3 (0.6)	2 (66.7)	7.77 (0.68 **to** 88.19)	
Not applicable/non-smoker	237 (50.3)	22 (9.28)	0.40 (0.22 **to** 0.71)	
Maternal age (years)				
Mean (SD)	26.5 (5.6)	24.6 (4.6)	0.93 (0.88 to 0.97)*	0.94 (0.89 to 1.00)†
Ethnicity				
White British	416 (88.9)	62 (14.9)	Reference†	Reference†
Other ethnicity	52 (11.1)	14 (26.9)	2.10 (1.08 to 4.11)	2.69 (1.19 to 6.06)
Highest qualification				
No qualifications	61 (13.0)	20 (32.8)	Reference*	
GCSEs or equivalent	183 (38.9)	28 (15.3)	0.37 (0.19 to 0.72)	
AS/A-Levels or equivalent	116 (24.6)	10 (8.6)	0.19 (0.08 to 0.45)	
Degree or equivalent	94 (20.0)	11 (11.7)	0.27 (0.12 to 0.62)	
Other qualification	17 (3.6)	7 (41.2)	1.43 (0.48 to 4.33)	
Age left full-time education				
≥17 years of age	208 (45.3)	22 (10.6)	Reference†	
≤16 years of age	230 (50.1)	48 (20.9)	2.23 (1.29 to 3.84)	
Still in full-time education	21 (4.6)	3 (14.3)	1.41 (0.38 to 5.17)	
Employment				
Paid work, manual	102 (21.8)	13 (12.8)	Reference	
Paid work, non-manual	129 (27.5)	13 (10.1)	0.77 (0.34 to 1.74)	
Paid work, unclear whether manual/non-manual	27 (5.8)	6 (22.2)	1.96 (0.67 to 5.75)	
Unemployed	90 (19.2)	21 (23.3)	2.08 (0.97 to 4.45)	
Full-time parent	97 (20.7)	18 (18.6)	1.56 (0.72 to 3.39)	
Full-time student	13 (2.8)	2 (15.4)	1.24 (0.25 to 6.26)	
Other	11 (2.4)	3 (27.27)	2.57 (0.60 to 10.93)	
Indices Multiple Deprivation score (IMD)‡				
1st tertile	157 (33.3)	16 (10.2)	Reference*	Reference†
2nd tertile	157 (33.3)	17 (10.8)	1.07 (0.52 to 2.20)	1.03 (0.47 to 2.25)
3rd tertile	157 (33.3)	43 (27.4)	3.32 (1.78 to 6.21)	2.30 (1.13 to 4.68)
Partner smoking at 3 months after childbirth				
Partner does not smoke tobacco	201 (42.7)	17 (8.5)	Reference*	
Partner smokes tobacco	220 (46.7)	51 (23.2)	3.27 (1.82 to 5.88)	
No partner	50 (10.6)	8 (16.0)	2.06 (0.83 to 5.09)	
Attitudes towards SHS				
Negative attitudes towards child SHS exposure (≥15 out of a possible 20)	419 (89.5)	51 (12.2)	Reference*	Reference*
Less negative attitudes towards child SHS exposure (≤14 out of a possible 20)	49 (10.5)	25 (51.0)	7.52 (4.00 to 14.14)	5.24 (2.57 to 10.68)

*Significant at p<0.001.

†Significant at p<0.05.

‡Higher score reflects greater deprivation.

GCSE, General Certificate of Secondary Education; SHS, secondhand smoke.

Table 2 shows the results of univariate analysis for factors associated with smoking in the home 3 months after childbirth, using non-imputed data. The strongest observed associations were for maternal smoking at 3 months after childbirth; those mothers smoking ≥11 cigarettes per day were 10.5 times more likely to report that smoking occurred in their home compared to non-smoking mothers at this time point. Maternal age, ethnicity, highest qualification, age left full-time education, IMD, partner smoking status and attitudes towards child SHS exposure score were also significantly associated with smoking in the home in univariate analysis.

In exploratory multivariable logistic regression modelling, smoking behaviour at 3 months after childbirth, younger maternal age, being of non-white British ethnicity, being more deprived as measured by IMD and holding less negative attitudes towards child SHS exposure were significantly associated with smoking in the home 3 months after childbirth (table 2). The strongest observed association was for mothers who smoked ≥11 cigarettes per day, who were over eight times more likely to report smoking occurred in their home.

## Discussion

After multiple imputation to control for non-response, the prevalence of smoking in the home at 3 months following childbirth was 18.2%. Prevalence was higher in homes where mothers who smoked lived compared to those where mothers were non-smokers (24% and 4.7%, respectively). Mothers who were currently smoking ≥11 cigarettes per day, younger, of non-white ethnicity, more deprived and held less negative attitudes towards child SHS exposure were significantly more likely to report that smoking occurred in their home 3 months after childbirth.

As far as we are aware, this is the first survey to investigate smoking in the home immediately after childbirth since the introduction of UK smoke-free legislation. Our estimate of the prevalence of SHS in the home was similar to estimates among slightly older infants from the USA, where 10.8–24.5% of infants of smoking mothers were exposed.^18^ ^19^ However, this was substantially lower than in the only previous UK survey in infants who were a similar age as those in our sample.^17^ In that study, 82% of infants aged on average 3 months old whose parents were smokers were exposed to SHS in the home. A number of factors are likely to have influenced our lower estimate of SHS exposure. Blackburn *et al*'s^17^ study was conducted in 2003; smoke-free legislations have since been implemented across the UK and this may have increased awareness of SHS and its implications. Additionally, UK smoking prevalence has reduced since the earlier survey, in particular among those of childbearing age^31^; increasing numbers of UK households are reported as smoke-free^2^ and older children's SHS exposure in the home has reduced.^5^ ^10^ Together these factors suggest that rates of smoking in the home will have declined since Blackburn's study.^17^

The observed prevalence of young infant's SHS exposure in the home is much lower than the most recent estimates of prevalence among older children in England, where 38.7% of children aged 4–15 years whose parents were smokers were exposed in the home in 2012.^6^ This finding is positive; young infants are particularly susceptible to the risks of SHS exposure as they have a higher respiration rate^32^ and underdeveloped lungs.^33^ This is exacerbated further as young infants experience increased SHS exposure due to the amount of time spent indoors in close proximity to smoking parents and surfaces such as carpets that have been contaminated with smoke, and having more hand to mouth contact compared to older children.^34^ However, SHS exposure is dangerous for children of all ages^2^; it is not yet known at what age parents or carers start to consider their children to be less vulnerable to the effects of SHS exposure and relax their home smoking restrictions. The early postnatal period, where the prevalence of SHS exposure in the home appears greatly reduced, may be a significant time-point to prevent future SHS exposure, before smoking in the home becomes an established behaviour.

In a recent systematic review,^20^ children whose parents were smokers, of low SES, less educated or held less negative attitudes towards SHS were at an increased risk of SHS exposure in the home, with the largest risks observed for children living in households with smokers. With the exception of parental education, the factors associated with young infant's SHS exposure in this study are similar to those among older children. The findings also show similarities to the current limited evidence base examining this in infants aged <2 years elsewhere; in the USA, having more children in the household, being of white ethnicity, low maternal education, low maternal age, being unmarried, lower income and markers of disadvantage during pregnancy were associated with infant SHS exposure.^18^ ^19^

A strength of this study was that during recruitment, 96% of women attending selected antenatal clinics within Nottingham University Hospital Trust were screened for eligibility, accounting for around one-third of all births within Nottingham, England, during this time.^23^ The demographic profile of smokers within this cohort is similar to the composition of other UK pregnancy cohorts,^23^ meaning that the sample is likely to be broadly representative. A potential limitation of this research was the lack of power within analysis due to small numbers of participants in some exposure variable groups. Furthermore, there were some differences between those who responded and those who did not respond at follow-up, which are described. These differences may have impacted on our prevalence estimates, however appropriate imputation methods were used to allow for this non-response bias. Non-response biases are less likely to have impacted on estimates of association with smoking in the home. A further potential limitation was reliance on reported smoking measures; parents may be inclined to give socially desirable responses resulting in under-estimates of children's SHS exposure.^35^ However, maternal-reported SHS exposure has been found to correlate with urinary cotinine and home environmental nicotine (r range 0.3–0.6) in infants aged <2.5 years.^36^ As the cohort included only women who were current or recent ex-smokers during pregnancy, the prevalence estimate obtained does not reflect children's SHS exposure in the home in the general population. However, as parental smoking, and in particular maternal smoking within the home, is the primary source of children and infant's SHS exposure,^20^ this study gives a useful indication of the scale of young infant's SHS exposure.

While the demographic characteristics associated with smoking in the home after childbirth are not easily modifiable, they may help to inform which infants, parents or families are best targeted in future interventions. The findings highlight that the best way to prevent or reduce smoking in the home immediately after childbirth is to help smoking mothers to quit and stay abstinent after childbirth. However, a recent systematic review did not find a significant effect of any behavioural intervention approach to prevent postpartum smoking relapse,^37^ and as such more research is needed to identify interventions which can support women at this important time. Where women are unable or unwilling to quit smoking, making their home smoke-free is the next most effective way to protect children.^38^ This study, consistent with research in older children,^20^ shows that less negative attitudes towards SHS exposure is associated with smoking in the home after childbirth. Interventions targeting attitudes towards SHS by supporting parents to recognise the benefits of protecting children from SHS may therefore be useful to promote smoke-free homes.

## Conclusions

The prevalence of smoking in homes where young infants live is lower than has been reported in older children (>3 months), suggesting that the early postnatal period may be an ideal time to intervene to prevent future SHS exposure in the home. The factors associated with smoking in the home immediately following childbirth were similar to those previously reported among older children. Interventions to support smoking mothers to quit, or to help them restrict smoking in the home, should target attitudinal change and address inequality relating to social disadvantage, younger age and non-white ethnic groups.
